# Genome-wide association study identifies new susceptibility loci for adolescent idiopathic scoliosis in Chinese girls

**DOI:** 10.1038/ncomms9355

**Published:** 2015-09-22

**Authors:** Zezhang Zhu, Nelson Leung-Sang Tang, Leilei Xu, Xiaodong Qin, Saihu Mao, Yueming Song, Limin Liu, Fangcai Li, Peng Liu, Long Yi, Jiang Chang, Long Jiang, Bobby Kin-Wah Ng, Benlong Shi, Wen Zhang, Jun Qiao, Xu Sun, Xusheng Qiu, Zhou Wang, Fei Wang, Dingding Xie, Ling Chen, Zhonghui Chen, Mengran Jin, Xiao Han, Zongshan Hu, Zhen Zhang, Zhen Liu, Feng Zhu, Bang-ping Qian, Yang Yu, Bing Wang, K. M. Lee, Wayne Y.W. Lee, T. P. Lam, Yong Qiu, Jack Chun-Yiu Cheng

**Affiliations:** 1 Department of Spine Surgery, the Affiliated Drum Tower Hospital of Nanjing University Medical School, Nanjing 210008, China; 2 Joint Scoliosis Research Center of The Chinese University of Hong Kong and Nanjing University, Nanjing 210008, China; 3 Department of Chemical Pathology, Faculty of Medicine, The Chinese University of Hong Kong, Hong Kong, China; 4 School of Biomedical Sciences, Faculty of Medicine, The Chinese University of Hong Kong, Hong Kong, China; 5 Li Ka Shing Institute of Health Sciences, Faculty of Medicine, The Chinese University of Hong Kong, Hong Kong, China; 6 Department of Orthopaedic, The West China Hospital, Sichuan University, Chengdu 610000, China; 7 Department of Orthopaedic, The Second Affiliated Hospital of Zhejiang University School of Medicine, Hangzhou 310000, China; 8 Department of Orthopaedic, China-Japan Union Hospital of Jilin University, Changchun 130022, China; 9 Jiangsu Key Laboratory for Molecular Medicine, Nanjing University Medical School, Nanjing 210008, China; 10 State Key Laboratory of Environment Health (Incubation), MOE (Ministry of Education) Key Laboratory of Environment & Health, Ministry of Environmental Protection Key Laboratory of Environment and Health (Wuhan), School of Public Health, Tongji Medical College, Huazhong University of Science and Technology, Wuhan 430000, China; 11 Department of Orthopaedic, Yixing People Hospital, Wuxi 214200, China; 12 Department of Orthopaedics and Traumatology, Faculty of Medicine, The Chinese University of Hong Kong, Hong Kong, China

## Abstract

Adolescent idiopathic scoliosis (AIS) is a structural deformity of the spine affecting millions of children. As a complex disease, the genetic aetiology of AIS remains obscure. Here we report the results of a four-stage genome-wide association study (GWAS) conducted in a sample of 4,317 AIS patients and 6,016 controls. Overall, we identify three new susceptibility loci at 1p36.32 near *AJAP1* (rs241215, *P*_combined_=2.95 × 10^−9^), 2q36.1 between *PAX3* and *EPHA4* (rs13398147, *P*_combined_=7.59 × 10^−13^) and 18q21.33 near *BCL-2* (rs4940576, *P*_combined_=2.22 × 10^−12^). In addition, we refine a previously reported region associated with AIS at 10q24.32 (rs678741, *P*_combined_=9.68 × 10^−37^), which suggests *LBX1AS1*, encoding an antisense transcript of *LBX1*, might be a functional variant of AIS. This is the first GWAS investigating genetic variants associated with AIS in Chinese population, and the findings provide new insight into the multiple aetiological mechanisms of AIS.

## Abstract (web summary)

The authors perform a genome-wide association study of adolescent idiopathic scoliosis patients of Han Chinese descent, and identify 3 new loci for disease susceptibility.

## Graphical abstract

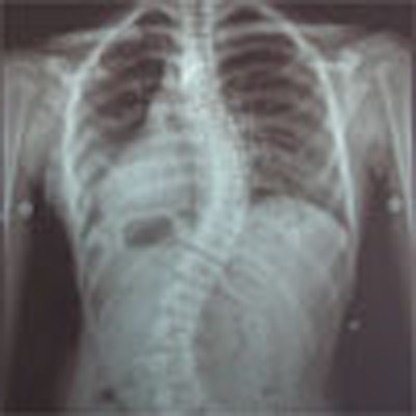

Adolescent idiopathic scoliosis (AIS) is a structural deformity of the spine that is estimated to affect millions of children, with a prevalence of 2–4% (refs 1, 2). Commonly understood as a complex trait (polygenic disease), the genetic aetiology of AIS has been widely investigated by linkage analysis and candidate gene association analysis^3^,^4^,^5^. During the era of candidate study, several genes were reported to be associated with AIS, such as *ERα* (ref. 6), *MATN1* (ref. 7), *MTNR1B* (ref. 8) and *TGFB1* (ref. 9). However, few of them have been successfully replicated in different ethnicities^10^,^11^,^12^,^13^. The first genome-wide association study (GWAS) in a Caucasian population reported that rs1400180 of *CHL1* was associated with AIS^14^, which however could not be replicated in a Chinese Han population^15^. Subsequently, a GWAS performed in a Japanese population revealed that *LBX1* and *GPR126* could play a role in the etiopathogenesis of AIS^16^,^17^. In a recent study that combined the GWAS data of AIS in Caucasian and Japanese populations, Sharma *et al.*^18^ reported an enhancer locus of *PAX1* was associated with the susceptibility of female AIS. As estimated by Kou *et al.*^17^, variants of *LBX1* and *GPR126* can only explain ∼1% of the trait variance in AIS. Herein additional genetic risk factors need to be discovered for a better understanding of the genetic susceptibility of AIS.

To map novel risk loci for AIS, here we report the results of a four-stage GWAS in a Chinese population. Overall, we identify three new susceptibility loci: 1p36.32 near *AJAP1*, encoding adherens junction associated protein 1 (rs241215, *P*_combined_=2.95 × 10^−9^); 2q36.1 between *PAX3* and *EPHA4*, encoding paired box 3 and EPH receptor A4, respectively (rs13398147, *P*_combined_=7.59 × 10^−13^); and 18q21.33 near *BCL-2*, encoding B-cell CLL/lymphoma 2 (rs4940576, *P*_combined_=2.22 × 10^−12^). In addition, we refine a previously reported region associated with AIS at 10q24.32 (rs678741, *P*_combined_=9.68 × 10^−37^), which suggests *LBX1AS1*, encoding an antisense transcript of *LBX1* (lady-bird like homeobox), might be the functional variant of AIS. This is the first GWAS to our knowledge investigating the genetic variants associated with AIS in a Chinese population, and the findings provide new insight into the multiple aetiological mechanisms of AIS.

## Results

### Association analyses in the discovery and replication

**Figure 1:**
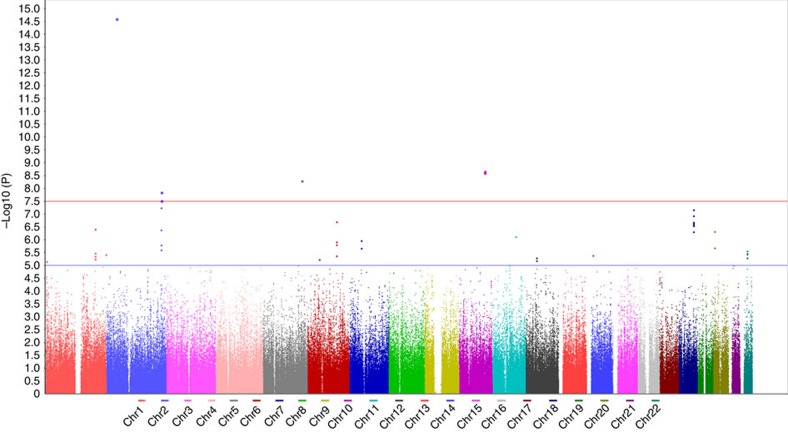
Genome-wide association results for AIS in Han Chinese girls. Here we present Manhattan plot of *P* values on the −log10 scale in the discovery stage (960 cases and 1,499 controls). The *x* axis shows Chromosomes as delineated by different colours. The *y* axis shows −log10 *P* values of each SNP. The red line represents *P*=5 × 10^−8^, and the blue dashed line represents *P*=1 × 10^−5^.

We performed a four-stage GWAS in cohorts of 4,317 patients and 6,016 controls, all of the Chinese Han population. In the discovery stage, 906,703 single nucleotide polymorphisms (SNPs) were genotyped in 1,001 patients presenting a main thoracic curve (major curve located in thoracic region) and 1,500 healthy controls. After quality control filtering of the genotyping data, 516,220 autosomal SNPs in 960 cases and 1,499 controls were retained for the subsequent Cochran–Armitage trend test. Analysis of population stratification with principal-component analysis (PCA) showed that all subjects of our study were clustered in Chinese population (Supplementary Fig. 1). The genomic inflation factor (*λ*) as shown by the quantile–quantile plot was 1.031, indicating that there was only a minimal amount of population stratification (Supplementary Fig. 2). As shown by the Manhattan plot (Fig. 1), 43 SNPs were observed to surpass the threshold of suggestive whole-genome significance (*P*<1.0 × 10^−5^) (Supplementary Table 2 and 3).

**Figure 2:**
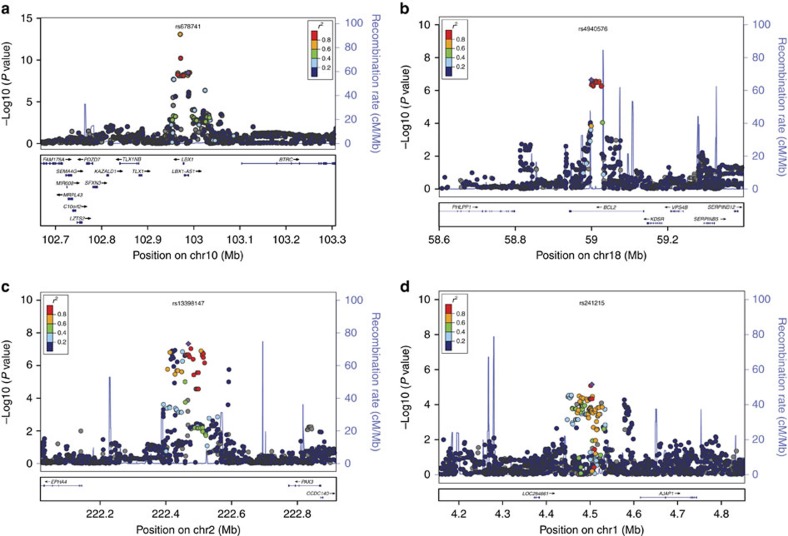
Regional association plots of four susceptibility loci for AIS. Each plot shows −log 10 (*P* value) for SNPs in the specific region. Imputation was performed by using 1000 Genomes Project CHB (Han Chinese in Beijing, China) data (November 2010 release) as a reference. The genotyped SNP with the strongest association signal in each locus is indicated by a purple diamond, and the other SNPs are coloured according to the *r*^2^ values with the proxy SNP. The genes within the regions of interest are annotated with the direction of transcription represented by arrows. (**a**) 10q24.32, (**b**) 18q21.33, (**c**) 2q36.1 and (**d**) 1p36.32.

From each putative haplotype block defined by linkage disequilibrium (*r*^2^> 0.8), we chose one representative SNP with the lowest *P* value for replication. Sixteen representative SNPs (Supplementary Table 2) were then included in replication stage 1, composed of 1,916 cases and 2,001 controls. Significant associations were found in four SNPs, which were further genotyped in 1,400 cases and 2,515 controls. Combining the results from the discovery stage and three replication stages, four SNPs were found to reach a genome-wide significance threshold of *P*<5 × 10^−8^, including rs241215 at 1p36, rs13398147 at 2q36, rs678741 at 10q24 and rs4940576 at 18p21 (Table 1). We performed imputation analysis around the four significant regions, and the logistic regression model showed similar association with those of the discovery stage (Supplementary Table 4). The strongest evidence of association was attained with rs678741 (*P*_combined_=9.68 × 10^−37^, odds ratio (OR)=1.44, 95% confidential interval (CI): 1.37–1.52, *P*_het_=0.95) located in the intron of the *LBX1AS1* (Table 1, Fig. 2a and Supplementary Fig. 3). SNP rs4940576 (*P*_combined_=2.22 × 10^−12^, OR=1.23, 95% CI: 1.14–1.29, *P*_het_=0.13) was located in the intronic region of *BCL-2* (Table 1, Fig. 2b and Supplementary Fig. 4). SNP rs13398147 (*P*_combined_=7.59 × 10^−13^, OR=1.28, 95% CI: 1.20–1.37, *P*_het_=0.11) was located 323 kb upstream of *EPHA4* and 304 kb downstream of *PAX3* (Table 1, Fig. 2c and Supplementary Fig. 5). SNP rs241215 (*P*_combined_=2.95 × 10^−9^, OR=0.83, 95% CI: 0.78–0.89, *P*_het_=0.32) maps 111 kb upstream of *AJAP1* (Table 1, Fig. 2d and Supplementary Fig. 6).

### Functional annotation

We evaluated the overlap of the novel associated SNPs with the Encyclopedia of DNA Elements (ENCODE)-annotated genomic elements^19^. Some signs of regulatory activity, including histone modifications at promoter or enhancer, DNase hypersensitivity sites, binding proteins and motifs changed, were observed for the four associated SNPs and their surrogates (Supplementary Tables 5–8).

## Discussion

In this four-stage GWAS, we identified four SNPs that are significantly associated with risk of AIS in the Chinese population. The linkage disequilibrium block of the top signal SNP (rs678741) at 10q24.32 contains a widely validated risk variant of AIS (rs11190870) mapping to the 3′ region of *LBX1* (refs 16, 20, 21, 22). Highly expressed in spinal cord and skeletal muscle, *LBX1* was identified as an AIS susceptibility gene by a previous GWAS^16^. *LBX1* may be implicated in the aetiology of scoliosis through abnormal somatosensory function^16^. In the current study, we further pinpointed a potentially regulatory SNP in this region. Data from ENCODE showed that rs678741 may be located in a strong enhancer region marked by peaks of several active histone methylation modifications. In addition, a search of HaploReg indicated that rs678741 maps to a DNase hypersensitivity site in multiple cell lines (Supplementary Table 5)^23^. These findings indicated that rs678741 has the potential to regulate the transcriptional activity of *LBX1AS1*. Previous studies have shown that antisense long non-coding RNA can regulate the expression of target genes by directly binding to the transcript^24^,^25^. Therefore, it is possible that *LBX1AS1* can be a biologically functional element that contributes to the risk of AIS. Further studies are necessary to clarify the role of *LBX1AS1* in the pathogenesis of AIS.

SNP rs4940576 is located in the intron of *BCL-2* gene that encodes an integral outer mitochondrial membrane protein playing a key role in apoptosis^26^,^27^. Amling *et al.*^28^ observed that *BCL-2* is expressed at highest levels in late proliferative and maturing chondrocytes. In *BCL-2* knockout mice, endochondral ossification was accelerated owing to the premature loss of terminal hypertrophic chondrocytes in the growth plate^29^. Thus, the product of *BCL-2* appears to have an important role in controlling osteoblast activity and regulating the endochondral-ossification process. Interestingly, overgrowth of anterior spinal endplate is a classic hypothesis in the etiopathogenesis of AIS^30^. Proliferative and hypertrophic chondrocytes in the anterior column of AIS patients seemed more active than that of the posterior column^31^. Moreover, AIS patients were found to have different growth kinetics in bilateral growth plate of the vertebrae as indicated by differences in cellular activity between the convex and concave side of the curve^32^.

The third SNP rs13398147 is located between *EPHA4* and *PAX3*. *EPHA4* belongs to the EPH receptor subfamily of the protein–tyrosine kinase family^33^. EPH receptors are implicated in mediating developmental events, particularly in the nervous system^34^. The *PAX3* subfamily regulates both myogenesis and neurogenesis in the neural tube^35^,^36^,^37^. The neural tube/notochord complex has a critical function during the development of vertebral muscle^38^. In addition, *PAX3* mutation can lead to muscular and neural tube defects, as well as malformation of the vertebral column^39^,^40^. Abnormality of the paravertebral muscles has been proposed as the cause of AIS^30^. Many diseases that affect muscle function can present a secondary scoliosis^41^,^42^.The fourth SNP rs241215 maps near *AJAP1*, encoding a transmembrane protein that interacts with E-cadherin/β-catenin complexes and the adaptor protein complex AP-1B in polarized epithelial cells^43^. Recent findings suggested a potential role for *AJAP1* in cell–cell and cell-extracellular matrix interactions that could be involved in cell adhesion, migration and invasion^44^,^45^. Regulation of cell adhesion has been found to be essential in the bone growth and osteoblast differentiation^46^,^47^,^48^.

To determine if there was any association between the novel associated SNPs and curve severity, we investigated a subgroup of 632 patients with a mean Cobb angle of 37.2±9.4° (range 27–49°). Of these, 214 patients received fusion surgery, and all other 418 who did not require surgical correction had been observed until skeletal maturity. As shown in Supplementary Table 9, rs241215 might be associated with the curve severity. Patients with genotype AA had a higher curve magnitude (39.6±7.3°) than those with genotype TA (36.9±7.7°) or TT (36.7±8.1°). As for the other three SNPs, no difference regarding the curve severity was found among the genotypes.

The primary limitation of our study lies in the lack of experiments supporting the functional role of the newly identified susceptibility genes. We believe that future functional analysis concerning the expression of these genes in tissues of AIS patients and related regulatory pathways can shed light on their relationships with the development of AIS. Moreover, future fine-mapping studies based on data from the resequencing of these regions may provide a clear understanding of the association at these loci.

In summary, our GWAS conducted in a Chinese Han population identifies three new susceptibility loci of AIS at 1p36.32, 2q36.1 and 18q21.33. This study also refines a region previously associated with AIS at 10q24.32, suggesting that *LBX1AS1*, an antisense transcript of *LBX1*, might be the functional variant of AIS. These findings provide additional insights into the genetic architecture of AIS. Further dissection of these loci is likely to expand our understanding of the aetiology of AIS.

## Methods

### Subjects

The current case-control analysis was composed of four stages, including the initial genome-wide discovery stage followed by three replication stages. The study has been approved by the following local Institutional Review Boards (IRB): IRB of Nanjing Drum Tower Hospital, IRB of The Chinese University of Hong Kong, IRB of The West China Hospital, IRB of The Second Affiliated Hospital of Zhejiang University and IRB of China-Japan Union Hospital of Jilin University. Patients who came to our joint scoliosis research clinics between 2000 and 2015 were reviewed for the eligibility to be included in this study. The inclusion criteria were as follows: (1) female; (2) diagnosed as AIS through clinical and radiologic examinations; (3) with Cobb angle >20°; and (4) having right major thoracic curve. Patients with scoliosis secondary to known aetiology, such as congenital scoliosis, neuromuscular scoliosis, scoliosis secondary to skeletal dysplasia and connective tissue abnormalities, were excluded from the study. Healthy subjects who came to our hospital for routine physical examinations were recruited as controls. Overall, there were 4,317 patients and 6,016 controls included in our study, all from ethnic Han Chinese population and collected in Nanjing of Jiangsu Province, Hangzhou of Zhejiang Province, Chengdu of Sichuan province, Changchun of Jilin Province and Hong Kong. The detailed profile of the subjects was as follows: in the discovery stage, 728 cases and 1,500 controls were collected in Nanjing, and the other 273 cases were collected in Hong Kong; in replication stage 1, all the 1,916 cases and 2,001 controls were collected in Nanjing; in replication stage 2, 307 cases and 1,735 controls were collected in Nanjing, and 600 cases and 180 controls were collected in Hong Kong, 15 cases were collected in Hangzhou, 10 cases were collected in Chengdu and 18 cases were collected in Changchun; in replications stage 3, all the 450 cases and 600 controls were collected in Nanjing. Under the approval of each participating centre's Ethical Committee, informed consent was obtained from all the participants and from the guardians of the adolescents. Clinical characteristics of the participants in the discovery stage were summarized in Supplementary Table 1.

### Genotyping and quality control for GWAS

Genomic DNA were extracted from peripheral blood leukocytes using a commercial DNA extraction kit (QIAGEN). Genotype analysis in the discovery stage was conducted using the Affymetrix Genome-Wide Human SNP Array 6.0 according to the manufacturer's protocol. Systemic quality control on the raw genotyping data in the discovery stage was carried out using PLINK (v1.90). The exclusion of unqualified SNPs was performed on the basis of following criteria: (1) with call rates <95%; (2) with minor allele frequency <0.05; (3) showing deviation from Hardy–Weinberg equilibrium (*P*<1 × 10^−5^); and (4) mapping on non-autosomal Chromosomes. For sample quality control, cryptic relatedness for each sample was evaluated with an identity-by-state method, and subjects showing second-degree relatedness or closer were removed (*n*=35). In addition, seven individuals with more than 10% missing genotypes were removed. There was no individual showing gender discrepancies based on their X chromosome genotypes. To detect population outliers and stratification, we used PCA implemented in the software package EIGENSTRAT^49^. A total of 2,459 participants remaining after sample quality control were analysed together with 210 HapMap subjects (60 YRI, 60 CEU, 45 JPT and 45 CHB individuals). Using the top two associated principal components, we identified that all individuals were of Chinese ancestry. Subsequently, we performed PCA using only the genotype information of the case and control subjects, and the scatterplot indicated that all cases and controls were genetically matched with minimal evidence of population stratification (Supplementary Fig. 1). After the quality control process, a total of 960 cases and 1,499 controls within Chinese Han population and 516,220 SNPs were used in the final analysis.

### Selection of SNPs for the replication stage

SNPs surpassing a threshold of suggestive genome-wide significance (*P*<1 × 10^−5^) in the discovery stage were potential candidates for replication. Among these SNPs, those with ambiguous genotype scatter plots were excluded from the replication stage. A total of 43 SNPs remained after filtering, from which we chose 16 representative SNPs for replication stage 1 and removed the other 27 SNPs due to high linkage disequilibrium with at least 1 of the 16 representative SNPs (Supplementary Table 2 and 3). After replication stage 1, 4 SNPs were included in replication stage 2 and 3, including rs678741, rs241215, rs13398147 and rs4940576 (Table 1 and Fig. 1). The SNP genotyping in replication stage 1 was performed with Sequenom MassARRAY system (Sequenom Inc.). Sixteen SNPs were multiplexed in one assay, and the primers were designed by custom software to give unique mass ranges for each SNP. Samples of replication stage 2 and 3 were genotyped using TaqMan SNP Genotyping Assay, which was read with an ABI Step-One-Plus sequence detection system (Applied Biosystems, Foster City, CA). Approximately 20 ng of genomic DNA was used to genotype each sample. Several quality control measures were applied as listed below. Laboratory technicians performing the genotyping assay were kept blind of case or control samples. In all, 5% of the samples were randomly selected as blind duplicates, with a reproducibility rate of 100%. DNA samples with >10% missing genotyping were excluded.

### Statistical analysis

In the discovery stage, a Cochran–Armitage trend test was used to calculate the association of each SNP with the disease. We calculated OR values and 95% CIs from a 2 × 2 allele frequency table. Data from the discovery stage and three replication stages were combined using the Mantel–Haenszel method. The heterogeneity between studies was examined using the Q-statistic *P* value^50^. The inflation factor *λ* was calculated by dividing the mean of the lower 90% of the test statistics by the mean of the lower 90% of the expected values from a *χ*^2^ distribution with 1 degree of freedom. We used PLINK 1.90 for general statistical analysis^51^. The Manhattan plot of −log10 *P* was generated using Haploview (v4.2)^52^. A quantile–quantile plot generated with R (v2.6) was used to evaluate the overall significance of the GWAS results and the potential impact of population stratification.

### Imputation

We performed genotype imputation within 800 kb around the four significant regions using MaCH-Admix software^53^. We used the linkage disequilibrium and haplotype information from the 1000 Genomes Project (phased CHB and CHS data; March 2012) as the reference set to impute ungenotyped markers (Supplementary Table 4). SNPs with minor allele frequency ≤0.05 or with a low imputation quality score (*R*^2^≤0.30) were excluded to ensure good imputation quality. Logistic regression analysis was used to determine the association of the SNPs with the AIS risk. The linkage disequilibrium pattern around the risk-associated SNPs was analysed to identify susceptibility genes that were covered by the linkage disequilibrium blocks. Linkage disequilibrium structures and haplotype block plots were generated with Haploview (v4.2)^52^. The four significant regions were plotted using the online tool LocusZoom^54^.

### Functional annotation

To explore the regulatory properties of the association signals, we used chromatin state segmentation in LCL data generated by the ENCODE project^19^. HaploReg was used to examine whether any of the SNPs or their proxies (that is, *r*^2^> 0.8 in the 1000 Genomes CHB reference panel) annotate putative transcription factor binding or enhancer elements (Supplementary Table 5-8)^23^.

### Subgroup analysis

To investigate the relationship between the novel associated SNPs and curve severity, two types of patients were included in the subgroup analysis: (1) being consistently observed until skeletal maturity; and (2) undergoing fusion surgery due to curve progression. Skeletal maturity was determined based on chronological age (≥16-years-old) or Risser sign (>4). The curve severity was recorded at the latest visit for patients being consistently observed, or at the last preoperative visit for those undergoing surgery. One-way analysis of variance test was used to compare the curve severity among different genotypes. Statistical significance was set at a *P* value of <0.05.

## Additional information

**How to cite this article:** Zhu, Z. *et al.* Genome-wide association study identifies new susceptibility loci for adolescent idiopathic scoliosis in Chinese girls. *Nat. Commun.* 6:8355 doi: 10.1038/ncomms9355 (2015).

## Supplementary Material

Supplementary Information Supplementary Figures 1-6 and Supplementary Tables 1-9

## Figures and Tables

**Table 1:** Association results for four SNPs in the GWAS, replication and combined samples.

**SNP**	**Chr.**	**Genes**	**MA**	**Stage**	**MAF**		***P*** **value**	**OR (95% CI)**	***P***_**het**_
					**Cases**	**Control**			
rs678741	10q24.32	*LBX1AS1*	A	GWAS	0.532	0.444	1.79 × 10^−9^	1.42 (1.27–1.60)	
				Replication 1	0.546	0.455	5.96 × 10^−14^	1.44 (1.32–1.58)	
				Replication 2	0.549	0.452	3.83 × 10^−12^	1.48 (1.32–1.66)	
				Replication 3	0.538	0.453	1.23 × 10^−4^	1.41 (1.18–1.68)	
				Combined	0.543	0.451	9.68 × 10^−37^	1.44 (1.37–1.52)	0.95
rs4940576	18q21.33	*BCL-2*	T	GWAS	0.471	0.393	7.01 × 10^−8^	1.38 (1.23–1.54)	
				Replication 1	0.419	0.384	2.06 × 10^−3^	1.16 (1.06–1.27)	
				Replication 2	0.430	0.386	1.68 × 10^−3^	1.20 (1.07–1.35)	
				Replication 3	0.431	0.382	2.37 × 10^−2^	1.23 (1.03–1.46)	
				Combined	0.435	0.387	2.22 × 10^−12^	1.23 (1.14–1.29)	0.13
rs13398147	2q36.1	*PAX3/EPHA4*	T	GWAS	0.267	0.198	1.47 × 10^−8^	1.48 (1.29–1.70)	
				Replication 1	0.233	0.201	9.59 × 10^−4^	1.20 (1.08–1.34)	
				Replication 2	0.239	0.202	1.39 × 10^−3^	1.24 (1.09–1.42)	
				Replication 3	0.241	0.202	3.38 × 10^−2^	1.25 (1.02–1.54)	
				Combined	0.243	0.201	7.59 × 10^−13^	1.28 (1.20–1.37)	0.11
rs241215	1p36.32	*AJAP1*	A	GWAS	0.261	0.321	7.55 × 10^−6^	0.75 (0.65–0.85)	
				Replication 1	0.299	0.329	5.49 × 10^−3^	0.87 (0.79–0.96)	
				Replication 2	0.291	0.323	7.09 × 10^−3^	0.85 (0.75–0.97)	
				Replication 3	0.284	0.327	3.71 × 10^−2^	0.82 (0.68–0.99)	
				Combined	0.287	0.325	2.95 × 10^−9^	0.83 (0.78–0.89)	0.32

Chr., chromosome; MA, minor allele; MAF, minor allele frequency; OR, odds ratio for the minor allele; *P*_het_, *P* value from the heterogeneity test based on GWAS and the two replication study groups.
