# Supplementary material for: Genome-wide association study identifies new susceptibility loci for adolescent idiopathic scoliosis in Chinese girls
Source: Nat Commun. 2015 Sep 22;6:8355. doi: 10.1038/ncomms9355 (PMC4595747; doi:10.1038/ncomms9355)
Supplement: Supplementary Information — Supplementary Figures 1-6 and Supplementary Tables 1-9 [file ncomms9355-s1.pdf]

Supplementary Figures

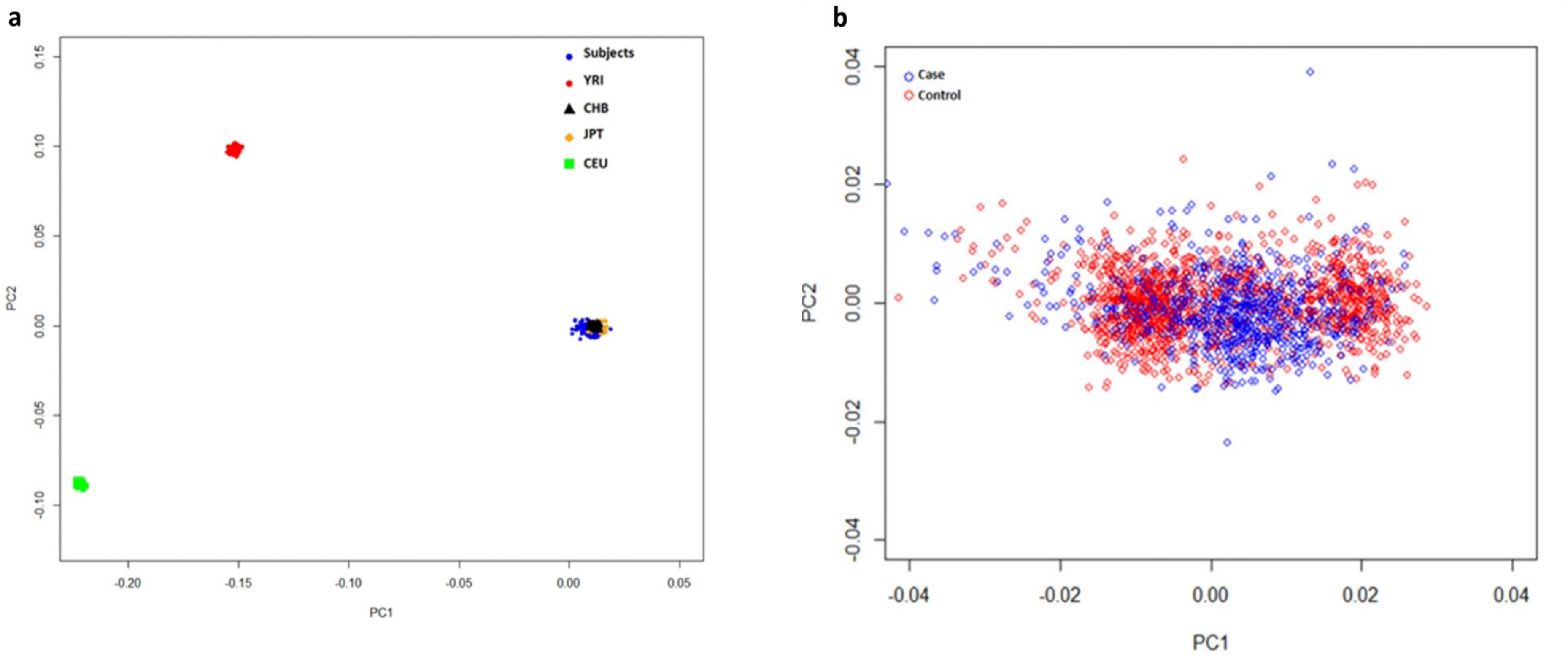

Supplementary Fig.1 Plot of the first two components derived from a principal component analysis

The figure shows that all the subjects are of Chinese ancestry. (a) Our samples & HapMap Phase-II data including European (CEU), African (YRI), Chinese (CHB), and Japanese (JPT); (b) Our samples(960 cases & 1499 controls).

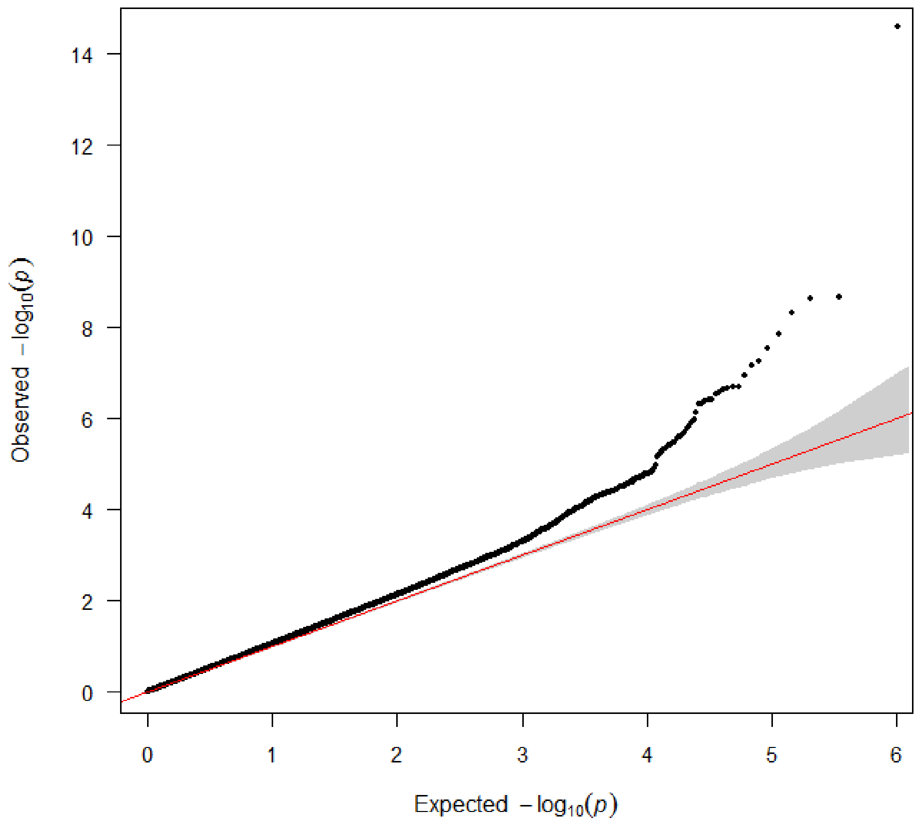

Supplementary Fig.2 Quantile-quantile plot of P-value distribution for SNPs in the discovery stage

The genomic inflation factor (λ) as shown by the quantile-quantile plot is 1.031, indicating a minimal amount of population stratification

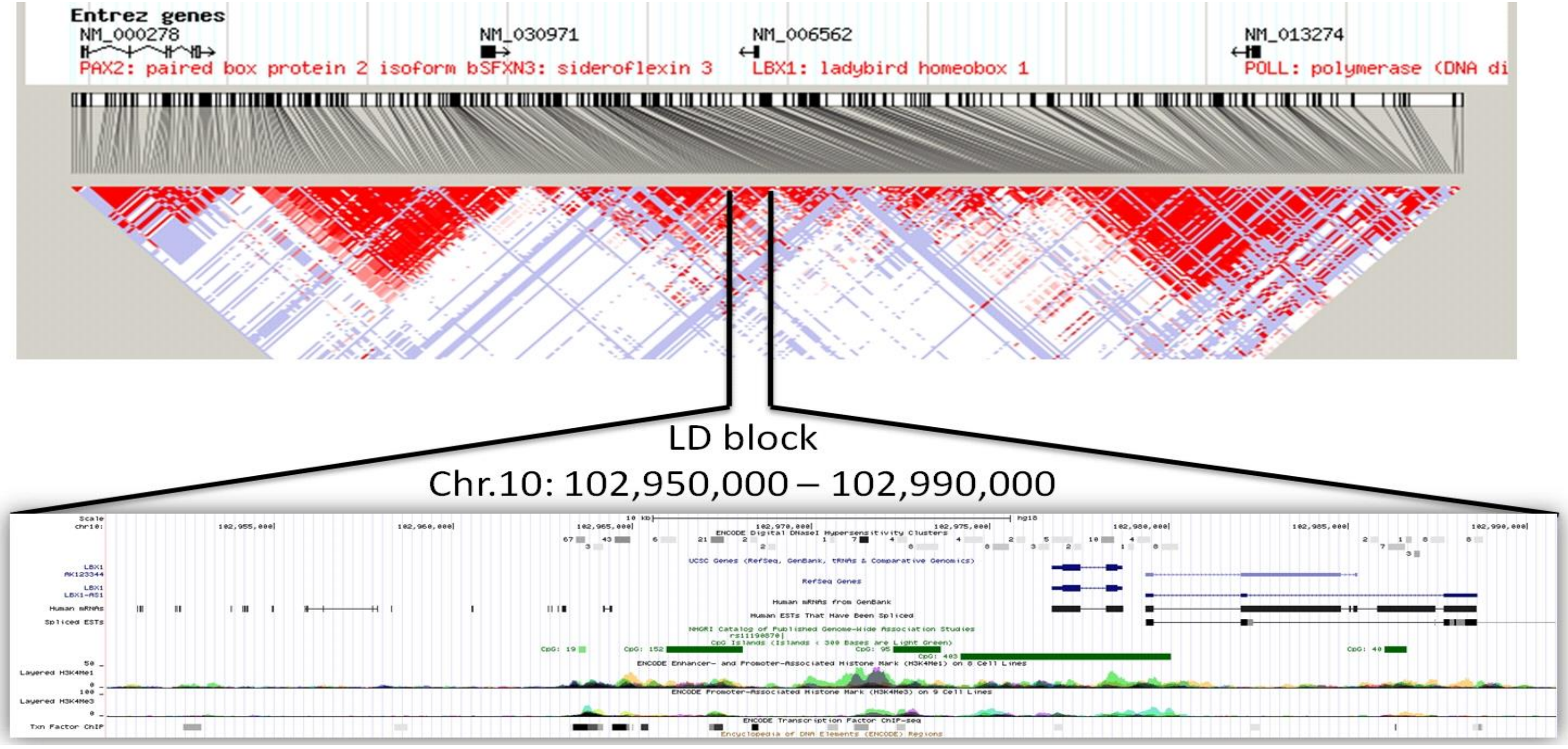

Supplementary Fig.3 Functional annotation in a linkage disequilibrium (LD) region containing the novel associated SNP rs678741 at 10q24 using UCSC genome browser

LD plot was generated in a window of 500 kb around the rs678741 based on HapMap III CHB population (release 2; accessed on Nov 08, 2012). For more visible figure, please visit: http://genome.ucsc.edu/cgibin/hgTracks?db=hg18&position=chr10%3A102950000102990000&hgsid=431542627_Sy0XgDmM5oV36B60IXVHrY8IjJb2

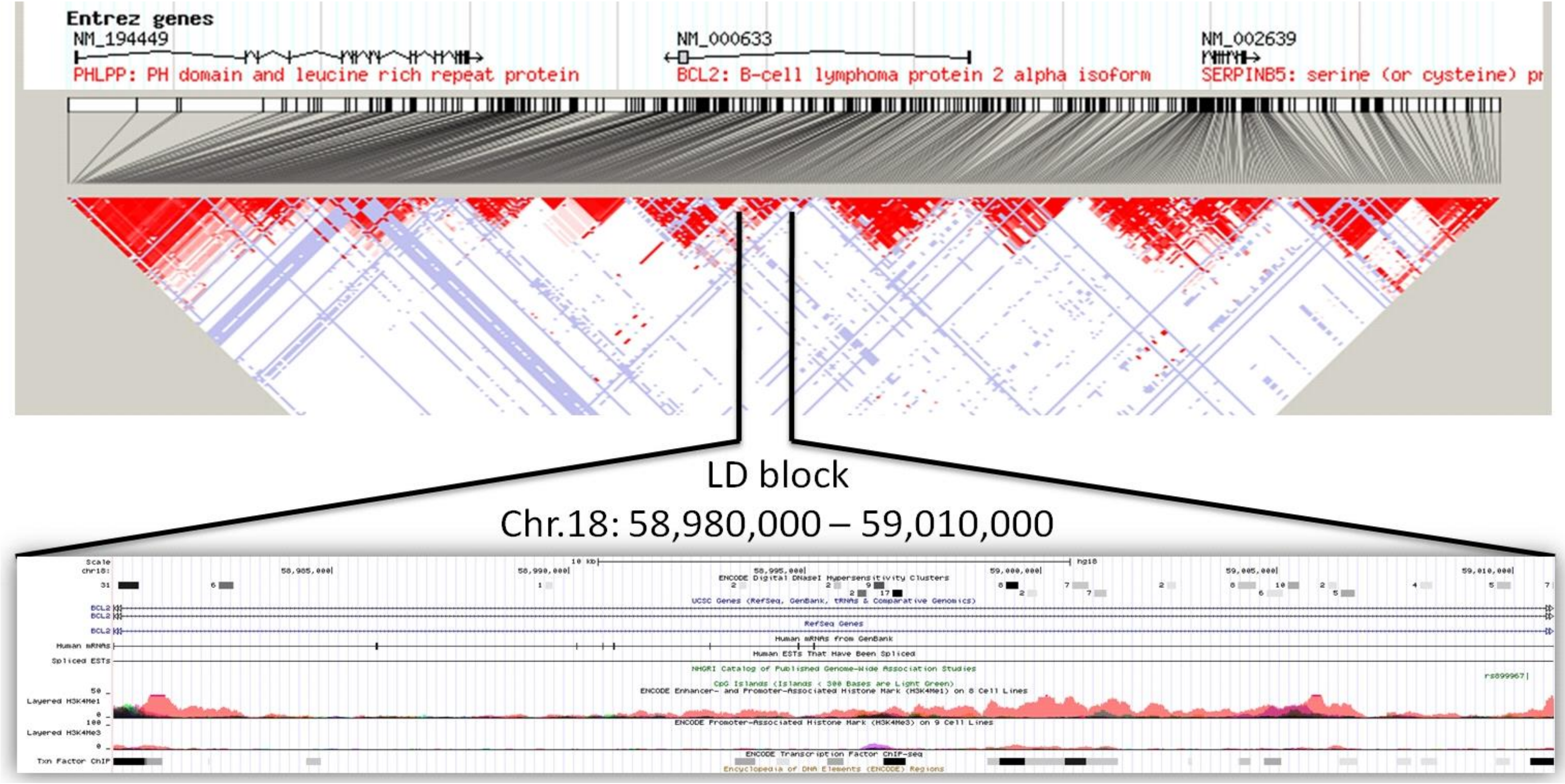

Supplementary Fig.4 Functional annotation in a linkage disequilibrium (LD) region containing the novel associated SNP rs4940576 at 18p21 using UCSC genome browser.

LD plot was generated in a window of 500 kb around the rs4940576 based on HapMap III CHB population (release 2; accessed on Nov 08, 2012). For more visible figure, please visit: http://genome.ucsc.edu/cgi-bin/hgTracks?db=hg18&position=chr18%3A58980000-59010000&hgsid=431542627_Sy0XgDmM5oV36B60IXVHrY8IjJb2

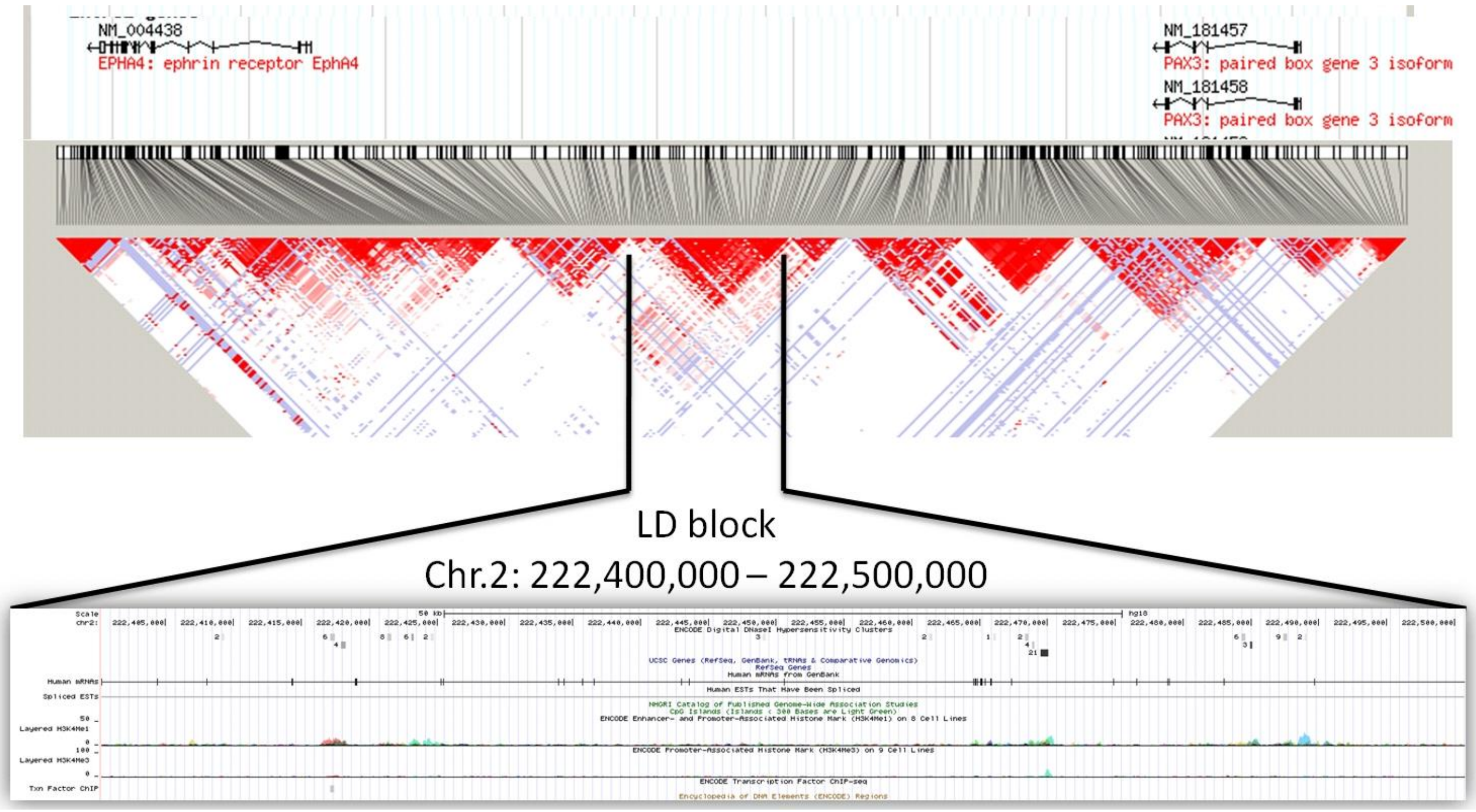

Supplementary Fig.5 Functional annotation in a linkage disequilibrium (LD) region containing the novel associated SNP rs13398147 at 2q36 using UCSC genome browser.

LD plot was generated in a window of 500 kb around the rs13398147 based on HapMap III CHB population (release 2; accessed on Nov 08, 2012). For more visible figure, please visit:
http://genome.ucsc.edu/cgi-bin/hgTracks?db=hg18&position=chr2%3A222400000-222500000&hgsid=431542627_Sy0XgDmM5oV36B60IXVHrY8IjJb2

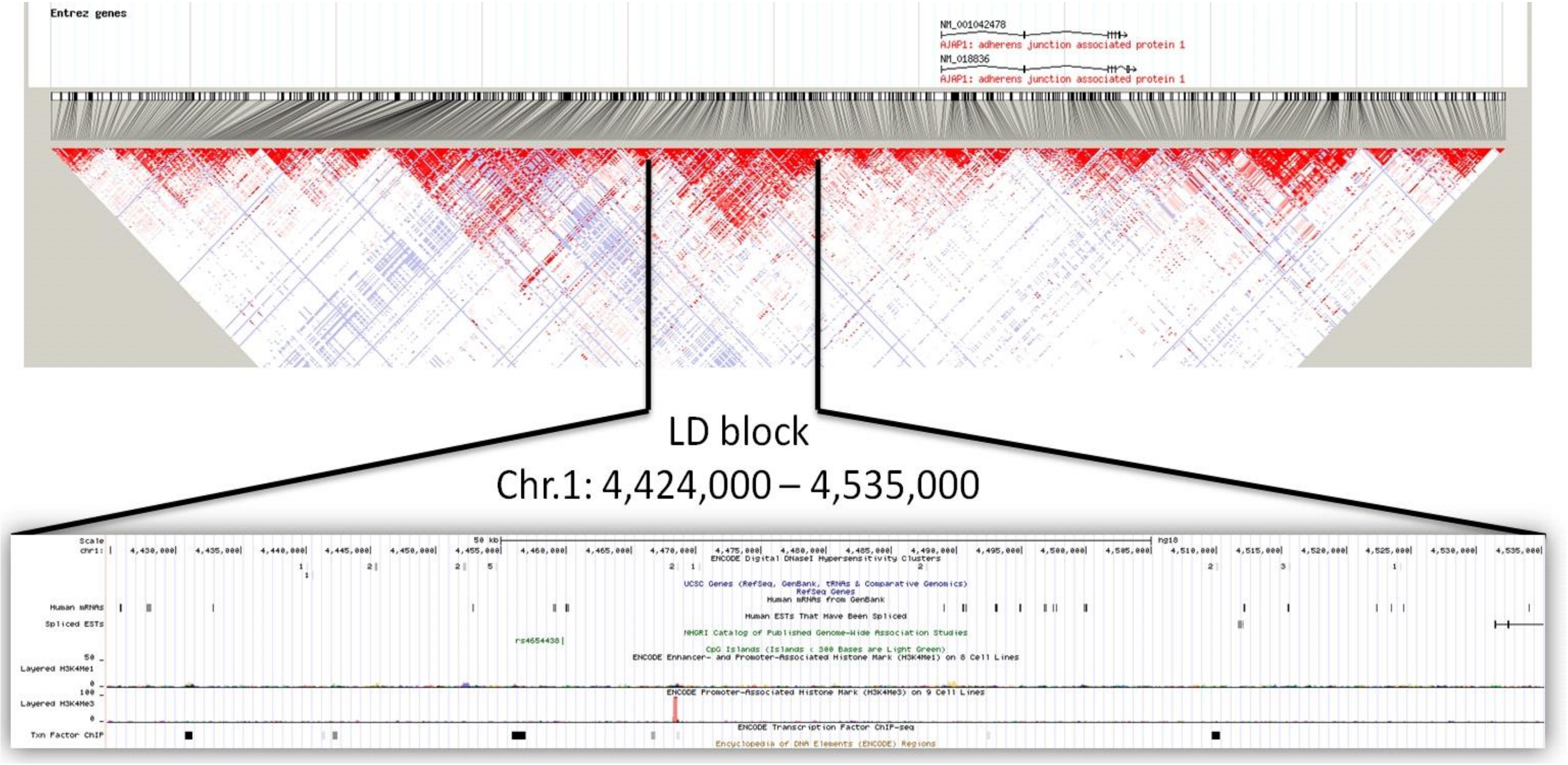

Supplementary Fig.6 Functional annotation in a linkage disequilibrium (LD) region containing the novel associated SNP rs241215 at 1p36 using UCSC genome browser.

LD plot was generated in a window of 500 kb around the rs241215 based on HapMap III CHB population (release 2; accessed on Nov 08, 2012). For more visible figure, please visit:
http://genome.ucsc.edu/cgi-bin/hgTracks?db=hg18&position=chr1%3A4424000-4535000&hgsid=431542627_Sy0XgDmM5oV36B60IXVHrY8IjJb2

Supplementary Tables

Supplementary Table 1 Clinical characteristics of the AIS patients in discovery stage

	Mean	Range
Age (years)	14.3 ± 3.2	10.2 - 17.5
Risser sign	4.2 ± 1.1	0 - 5
Curve magnitude (degrees)	38.5 ± 12.3	21 - 67
Year post-menarche (years)	3.1 ± 1.7	0 - 4.4
Body mass index (kg/m^2)	17.9 ± 3.8	16.7 - 22.8

Supplementary Table 2 Results for the 16 replicated SNPs analyzed in the discovery stage and replication stage 1

SNP	CHR.	MA	MAF†		P†	OR† (95% CI)	MAF††		P††	OR†† (95% CI)
			Cases	Controls			Cases	Controls		
rs241215	1	T	0.261	0.321	7.55×10^{-6}	0.75 (0.65-0.85)	0.299	0.329	5.49×10^{-3}	0.87 (0.79-0.96)
rs2096196	1	T	0.221	0.169	5.63×10^{-6}	1.40 (1.21-1.61)	0.207	0.194	0.16	1.09 (0.97-1.21)
rs7366357	1	C	0.238	0.299	3.39×10^{-6}	0.73 (0.64-0.83)	0.285	0.296	0.30	0.95 (0.86-1.05)
rs1544818	2	A	0.251	0.158	5.13×10^{-15}	1.79 (1.55-2.07)	0.252	0.234	0.07	1.01 (0.99-1.21)
rs13398147	2	T	0.267	0.198	1.47×10^{-8}	1.48 (1.29-1.70)	0.233	0.201	9.59×10^{-4}	1.20 (1.08-1.34)
rs17643057	5	G	0.262	0.190	9.13×10^{-9}	1.51 (1.31-1.73)	0.265	0.254	0.28	1.06 (0.96-1.17)
rs11966702	6	C	0.174	0.127	6.28×10^{-6}	1.45 (1.23-1.70)	0.163	0.150	0.12	1.10 (0.97-1.25)
rs17079281	6	A	0.266	0.331	1.16×10^{-6}	0.73 (0.65-0.83)	0.312	0.325	0.23	0.94 (0.86-1.04)
rs10248242	7	A	0.529	0.458	1.14×10^{-6}	1.33 (1.19-1.49)	0.488	0.475	0.26	1.05 (0.96-1.15)
rs678741	10	T	0.532	0.444	1.79×10^{-9}	1.42 (1.27-1.60)	0.546	0.455	5.96×10^{-14}	1.44 (1.32-1.58)
rs11822727	11	A	0.351	0.283	7.96×10^{-7}	1.37 (1.21-1.55)	0.297	0.287	0.34	1.05 (0.95-1.16)
rs7956314	12	G	0.347	0.285	3.91×10^{-6}	1.33 (1.18-1.51)	0.337	0.328	0.41	1.04 (0.95-1.15)
rs10483313	14	A	0.162	0.216	2.08×10^{-6}	0.70 (0.60-0.82)	0.196	0.205	0.33	0.95 (0.84-1.06)
rs4940576	18	A	0.471	0.393	7.01×10^{-8}	1.38 (1.23-1.54)	0.419	0.384	2.06×10^{-3}	1.16 (1.06-1.27)
rs6054258	20	T	0.297	0.367	5.62×10^{-7}	0.73 (0.64-0.82)	0.348	0.362	0.21	0.94 (0.86-1.03)
rs134636	22	T	0.274	0.216	3.90×10^{-6}	1.37 (1.20-1.57)	0.264	0.248	0.11	1.09 (0.98-1.21)

CHR., chromosome; MA, minor allele; MAF, minor allele frequency; OR, odds ratio for the minor allele; 95% CI, 95% confidence intervals; †, discovery stage; ††, replications stage 1

Supplementary Table 3 Results for the 27 SNPs that were removed from replication stage due to high LD with at least 1 of the 16 representative SNPs

SNP	CHR.	MA	MAF		P	OR (95%CI)
			Cases	Controls		
rs10494848	1	A	0.226	0.167	3.74×10^{-7}	1.46 (1.26-1.68)
rs2886616	1	T	0.223	0.170	4.38×10^{-6}	1.40 (1.21-1.62)
rs13374988	1	T	0.220	0.166	3.28×10^{-6}	1.41 (1.22-1.63)
rs3905172	1	A	0.226	0.167	3.93×10^{-7}	1.45 (1.26-1.68)
rs7580262	2	G	0.264	0.205	1.59×10^{-6}	1.39 (1.22-1.59)
rs4085399	2	A	0.264	0.207	2.48×10^{-6}	1.38 (1.21-1.58)
rs17312518	2	T	0.264	0.207	2.48×10^{-6}	1.38 (1.21-1.58)
rs17312814	2	T	0.254	0.193	4.04×10^{-7}	1.43 (1.24-1.63)
rs4099423	2	C	0.258	0.193	5.70×10^{-8}	1.46 (1.27-1.67)
rs824918	2	A	0.244	0.179	2.91×10^{-8}	1.48 (1.29-1.71)
rs2104064	6	G	0.267	0.332	1.19×10^{-6}	0.73 (0.64-0.83)
rs7763979	6	T	0.289	0.353	4.17×10^{-6}	0.75 (0.66-0.85)
rs13210963	6	A	0.266	0.337	1.96×10^{-7}	0.71 (0.63-0.81)
rs10261964	7	T	0.529	0.460	2.14×10^{-6}	1.32 (1.18-1.48)
rs10883597	10	G	0.526	0.438	2.59×10^{-9}	1.42 (1.27-1.59)
rs9706460	12	A	0.346	0.285	6.58×10^{-6}	1.33 (1.17-1.50)
rs9962656	18	A	0.466	0.393	4.77×10^{-7}	1.35 (1.20-1.51)
rs1531695	18	C	0.472	0.395	1.17×10^{-7}	1.37 (1.22-1.54)
rs4941187	18	C	0.472	0.398	2.81×10^{-7}	1.35 (1.21-1.52)
rs12968867	18	C	0.475	0.399	2.04×10^{-7}	1.36 (1.21-1.53)
rs4941188	18	G	0.470	0.395	2.69×10^{-7}	1.36 (1.21-1.52)
rs899966	18	T	0.469	0.394	2.15×10^{-7}	1.36 (1.21-1.53)
rs12457700	18	T	0.469	0.395	2.34×10^{-7}	1.36 (1.21-1.52)
rs6054251	20	G	0.300	0.366	2.01×10^{-6}	0.74 (0.66-0.84)

rs713861	22	G	0.274	0.216	3.36×10^{-6}	1.37 (1.20-1.56)
rs134653	22	T	0.274	0.217	3.61×10^{-6}	1.37 (1.20-1.56)
rs134666	22	G	0.280	0.223	4.97×10^{-6}	1.36 (1.19-1.55)

CHR., chromosome; MA, minor allele; MAF, minor allele frequency; OR, odds ratio for the minor allele; 95% CI, 95% confidence intervals

Supplementary Table 4 Results for the genotyped or imputed SNPs at 1p36.3, 2q36.1, 10q24.3, and 18p21.3 that were associated with AIS ($p<1.0 \times 10^{-5}$)

CHR.	SNP	Position	MAF		P†	OR (95%CI)	LD(r^2)	Identification
			Cases	Controls				
**1**	**rs241215**	**4604104**	**0.26**	**0.32**	**7.08×10^{-6}**	**0.75 (0.66-0.85)**	**1**	**Genotyping**
1	rs627374	4600233	0.26	0.32	8.44×10^{-6}	0.75 (0.66-0.86)	0.96	Imputation
**2**	**rs13398147**	**222760279**	**0.26**	**0.20**	**4.66×10^{-8}**	**1.46 (1.28-1.67)**	**1**	**Genotyping**
2	rs4099423	222766109	0.26	0.19	9.58×10^{-8}	1.45 (1.27-1.67)	0.94	Genotyping
2	rs17312308	222702714	0.27	0.20	1.61×10^{-7}	1.48 (1.29-1.69)	0.84	Imputation
2	rs824929	222801432	0.24	0.18	1.65×10^{-7}	1.45 (1.26-1.67)	0.73	Imputation
2	rs824927	222800429	0.24	0.18	1.79×10^{-7}	1.46 (1.26-1.68)	0.75	Imputation
2	rs824934	222804226	0.23	0.17	1.96×10^{-7}	1.46 (1.27-1.68)	0.72	Imputation
2	rs17312759	222755728	0.26	0.19	2.23×10^{-7}	1.44 (1.26-1.65)	0.96	Imputation
2	rs17312814	222758822	0.26	0.19	2.23×10^{-7}	1.44 (1.26-1.65)	0.96	Genotyping
2	rs4674621	222759056	0.26	0.19	2.23×10^{-7}	1.44 (1.26-1.65)	0.96	Imputation
2	rs62180527	222752116	0.26	0.19	2.25×10^{-7}	1.44 (1.25-1.65)	0.95	Imputation
2	rs824928	222800746	0.24	0.18	2.53×10^{-7}	1.45 (1.26-1.67)	0.73	Imputation
2	rs17312891	222762474	0.26	0.19	2.98×10^{-7}	1.43 (1.25-1.64)	0.95	Imputation
2	rs62180530	222761682	0.26	0.19	2.98×10^{-7}	1.43 (1.25-1.64)	0.96	Imputation
2	rs7567306	222761862	0.26	0.19	2.98×10^{-7}	1.43 (1.25-1.64)	0.96	Imputation
2	rs824933	222804191	0.23	0.17	3.36×10^{-7}	1.45 (1.26-1.67)	0.72	Imputation
2	rs6739602	222772976	0.24	0.18	4.20×10^{-7}	1.48 (1.29-1.71)	0.77	Imputation
2	rs7580262	222695346	0.26	0.21	1.74×10^{-6}	1.39 (1.22-1.59)	0.84	Genotyping
2	rs62180487	222723462	0.26	0.21	2.11×10^{-6}	1.39 (1.21-1.59)	0.86	Imputation
2	rs4510218	222726388	0.26	0.21	2.11×10^{-6}	1.39 (1.21-1.59)	0.86	Imputation
2	rs77667659	222805620	0.23	0.18	2.21×10^{-6}	1.40 (1.22-1.62)	0.7	Imputation
2	rs72971048	222730890	0.26	0.21	2.48×10^{-6}	1.38 (1.21-1.58)	0.86	Imputation
2	rs4085399	222737884	0.26	0.21	2.48×10^{-6}	1.38 (1.21-1.58)	0.87	Genotyping

2	rs17312518	222738041	0.26	0.21	2.48×10^{-6}	1.38 (1.21-1.58)	0.87	Genotyping
2	rs62180522	222738423	0.26	0.21	2.48×10^{-6}	1.38 (1.21-1.58)	0.87	Imputation
2	rs824922	222789037	0.24	0.18	2.81×10^{-6}	1.49 (1.30-1.72)	0.75	Imputation
2	rs824923	222789106	0.24	0.18	2.81×10^{-6}	1.49 (1.30-1.72)	0.75	Imputation
2	rs824918	222785645	0.24	0.18	2.81×10^{-6}	1.49 (1.30-1.72)	0.76	Genotyping
2	rs824920	222786280	0.24	0.18	2.81×10^{-6}	1.49 (1.30-1.72)	0.76	Imputation
2	rs4674617	222717946	0.26	0.19	3.51×10^{-6}	1.54 (1.34-1.76)	0.84	Imputation
2	rs62180532	222775058	0.24	0.18	3.88×10^{-6}	1.49 (1.29-1.71)	0.77	Imputation
2	2:222798138:AT_A	222798138	0.24	0.18	3.92×10^{-6}	1.49 (1.29-1.71)	0.76	Imputation
10	rs11190870	102979207	0.37	0.48	8.68×10^{-14}	0.64 (0.57-0.72)	0.65	Imputation
10	rs11598177	102980156	0.53	0.44	6.35×10^{-11}	1.44 (1.28-1.61)	0.97	Imputation
**10**	**rs678741**	**102997581**	**0.53**	**0.44**	**3.28×10^{-9}**	**1.43 (1.27-1.60)**	**1**	**Genotyping**
10	rs10883597	102999754	0.53	0.44	3.45×10^{-9}	1.42 (1.27-1.60)	0.94	Genotyping
10	rs3950032	102974109	0.32	0.42	3.83×10^{-9}	0.67 (0.60-0.76)	0.5	Imputation
10	rs1322330	102991659	0.32	0.41	4.25×10^{-9}	0.66 (0.58-0.74)	0.54	Imputation
10	rs679206	102997450	0.53	0.44	4.43×10^{-9}	1.43 (1.27-1.60)	0.99	Imputation
10	rs1535462	102973872	0.52	0.44	5.95×10^{-9}	1.40 (1.25-1.57)	0.94	Imputation
10	rs1407409	102985407	0.32	0.42	6.25×10^{-9}	0.66 (0.58-0.74)	0.53	Imputation
10	rs594791	102995796	0.37	0.47	7.09×10^{-9}	0.65 (0.58-0.73)	0.69	Imputation
10	rs76319884	102976006	0.32	0.42	7.20×10^{-9}	0.66 (0.58-0.74)	0.5	Imputation
10	rs1322331	102986589	0.53	0.44	8.00×10^{-9}	1.43 (1.27-1.60)	0.98	Imputation
10	rs1322332	102982648	0.52	0.44	8.31×10^{-9}	1.43 (1.27-1.60)	0.96	Imputation
10	rs7914775	102976661	0.32	0.42	8.40×10^{-9}	0.66 (0.58-0.74)	0.52	Imputation
10	rs11598564	102964604	0.33	0.40	2.15×10^{-8}	0.75 (0.66-0.84)	0.25	Imputation
10	rs12771674	102965309	0.37	0.44	2.28×10^{-8}	0.73 (0.65-0.83)	0.3	Imputation
10	rs79648198	102983088	0.28	0.37	5.50×10^{-8}	0.68 (0.60-0.76)	0.46	Imputation
10	rs7919667	102968878	0.39	0.33	2.48×10^{-6}	1.33 (1.18-1.49)	0.23	Imputation
**18**	**rs4940576**	**60848639**	**0.47**	**0.40**	**1.80×10^{-7}**	**1.36 (1.21-1.53)**	**1**	**Genotyping**

18	rs12968867	60850310	0.47	0.40	2.14×10^{-7}	1.36 (1.21-1.52)	0.98	Genotyping
18	rs899966	60859241	0.47	0.39	2.34×10^{-7}	1.36 (1.21-1.52)	0.9	Genotyping
18	rs899967	60859321	0.47	0.39	2.34×10^{-7}	1.36 (1.21-1.52)	0.9	Imputation
18	rs744569	60859507	0.47	0.39	2.34×10^{-7}	1.36 (1.21-1.52)	0.9	Imputation
18	rs731014	60859509	0.47	0.39	2.34×10^{-7}	1.36 (1.21-1.52)	0.9	Imputation
18	rs12454650	60867780	0.47	0.39	2.50×10^{-7}	1.36 (1.21-1.52)	0.9	Imputation
18	rs1531695	60848823	0.47	0.40	2.60×10^{-7}	1.36 (1.21-1.52)	0.98	Genotyping
18	rs4941187	60849216	0.47	0.40	2.60×10^{-7}	1.36 (1.21-1.52)	0.98	Genotyping
18	rs9944895	60859974	0.47	0.39	2.83×10^{-7}	1.35 (1.21-1.52)	0.9	Imputation
18	rs12457700	60860246	0.47	0.39	2.83×10^{-7}	1.35 (1.21-1.52)	0.9	Genotyping
18	rs12962650	60860569	0.47	0.39	2.83×10^{-7}	1.35 (1.21-1.52)	0.9	Imputation
18	rs12958785	60860642	0.47	0.39	2.83×10^{-7}	1.35 (1.21-1.52)	0.9	Imputation
18	rs58807442	60860890	0.47	0.39	2.83×10^{-7}	1.35 (1.21-1.52)	0.9	Imputation
18	rs11152372	60860930	0.47	0.39	2.83×10^{-7}	1.35 (1.21-1.52)	0.9	Imputation
18	rs4987790	60861693	0.47	0.39	2.83×10^{-7}	1.35 (1.21-1.52)	0.9	Imputation
18	rs4941188	60857029	0.47	0.40	3.01×10^{-7}	1.35 (1.21-1.52)	0.9	Genotyping
18	rs8098848	60857793	0.47	0.40	3.01×10^{-7}	1.35 (1.21-1.52)	0.9	Imputation
18	rs11875971	60867576	0.47	0.39	3.03×10^{-7}	1.35 (1.21-1.52)	0.9	Imputation
18	rs4987792	60851880	0.47	0.40	3.54×10^{-7}	1.35 (1.20-1.52)	0.95	Imputation
18	rs12457371	60847097	0.47	0.39	3.68×10^{-7}	1.35 (1.20-1.52)	0.97	Imputation
18	rs9962656	60847262	0.47	0.39	3.68×10^{-7}	1.35 (1.20-1.52)	0.97	Genotyping
18	rs11661511	60852258	0.47	0.40	3.82×10^{-7}	1.35 (1.20-1.51)	0.91	Imputation
18	rs113237723	60860860	0.47	0.40	4.64×10^{-7}	1.35 (1.20-1.51)	0.9	Imputation
18	rs1481031	60852085	0.47	0.40	4.79×10^{-7}	1.35 (1.20-1.51)	0.91	Imputation
18	rs960804	60874418	0.46	0.39	9.62×10^{-7}	1.34 (1.19-1.50)	0.85	Imputation
18	rs2062010	60873082	0.46	0.39	1.17×10^{-6}	1.33 (1.19-1.50)	0.85	Imputation

The imputed SNPs were imputed using MaCH-Admix software with LD and haplotype information from the 1000Genome Project March 2012 release CHB+JPT population as the reference. CHR., chromosome; MA, minor allele; MAF, minor allele frequency; OR, odds ratio for the minor

allele; 95% CI, 95% confidence intervals; LD, linkage disequilibrium; † *P* values are two-sided and were calculated by an additive model in logistic regression analysis

Supplementary Table 5 Functional annotation of SNPs correlated with rs678741 (r2 > 0.8) using data from ENCODE

Pos. (hg38)	LD (r^2)[a]	SNP[b]	Histone marks		DNAse[c]	Motifs changed[d]	Genes	Annotation
			Promoter	Enhancer				
Chr.10:101214115	0.88	rs1535462	15 organs	18 organs	12 organs	4 altered motifs	13kb 3' of LBX1	
Chr.10:101220399	0.91	rs11598177	IPSC	BRST, SKIN		5 altered motifs	6.6kb 3' of LBX1	
Chr.10:101222891	0.9	rs1322332	15 organs	18 organs	19 organs	Ik-2,PTF1-beta	4.1kb 3' of LBX1	
Chr.10:101226832	0.91	rs1322331	7 organs	17 organs	21 organs	15 altered motifs	143bp 3' of LBX1	
Chr.10:101237693	1	rs679206	ESC, IPSC, FAT	8 organs	28 organs	GATA	LBX1AS1	intronic
**Chr.10:101237824**	**1**	**rs678741**	**ESC, IPSC, FAT**	**8 organs**	**29 organs**		**LBX1AS1**	**intronic**
Chr.10:101239997	0.87	rs10883597			ESDR,BLD,BLD	Bcl6b,EBF,STAT	5' of LBX1AS1	

ENCODE, the Encyclopedia of DNA Elements; LD, linkage disequilibrium; Chr., Chromosome; Pos., Position

[a] LD (r^2) is based upon 1000 Genomes data and a threshold of r^2>0.8 was imposed to identify correlated SNPs

[b] Index SNPs are shown in bold.

[c] Evidence of Chromatin hypersensitivity to DNase

[d] Evidence of alteration in regulatory motif (if >3, only the number is included).

Supplementary Table 6 Functional annotation of SNPs correlated with rs4940576 (r^2 > 0.8) using data from ENCODE

Pos. (hg38)	LD (r^2)[a]	SNP[b]	Histone marks		DNAse[c]	Proteins Bound[d]	Motifs changed[e]	Genes	Annotation
			Promoter	Enhancer					
Chr.18:63179197	0.84	rs74625348	BRST, BRN, MUS	17 organs	7 organs		GLI,Klf4,NRSF	BCL2	intronic
Chr.18:63179423	0.9	rs80030866	BRST, BRN, MUS	16 organs	ESC,MUS		5 altered motifs	BCL2	intronic
Chr.18:63179505	0.91	rs17070809	BRST, BRN, MUS	16 organs	ESDR,ESC		Mef2	BCL2	intronic
Chr.18:63180029	0.87	rs9962656	BRN	10 organs			Irf,Mef2	BCL2	intronic
**Chr.18:63181406**	**1**	**rs4940576**	**BLD**	**10 organs**	**BRN,MUS,BLD**	**POL2**	**BDP1**	**BCL2**	**intronic**
Chr.18:63181590	0.84	rs1531695		9 organs			HDAC2,NRSF	BCL2	intronic
Chr.18:63181983	0.83	rs4941187	GI	11 organs	ESDR,GI	POL2,POL24H8	NF-kappaB,VDR	BCL2	intronic
Chr.18:63183077	0.84	rs12968867	GI, BLD	15 organs	12 organs		BDP1	BCL2	intronic
Chr.18:63184647	0.82	rs4987792		6 organs	ESC,BLD		NF-Y	BCL2	intronic
Chr.18:63185025	0.82	rs11661511		7 organs	ESC,MUS			BCL2	intronic
Chr.18:63189796	0.82	rs4941188		4 organs	IPSC		GR,HNF1,STAT	BCL2	intronic
Chr.18:63190560	0.82	rs8098848		BLD	BLD		5 altered motifs	BCL2	intronic
Chr.18:63192008	0.82	rs899966		4 organs	HRT		Bbx,Hbp1,VDR	BCL2	intronic
Chr.18:63192088	0.82	rs899967		4 organs	ESDR,BLD,SKIN		4 altered motifs	BCL2	intronic
Chr.18:63192274	0.82	rs744569		7 organs	BLD,BLD		Nkx2	BCL2	intronic
Chr.18:63192276	0.82	rs731014		7 organs	BLD,BLD		Irf	BCL2	intronic
Chr.18:63192741	0.85	rs9944895	MUS	9 organs	11 organs		13 altered motifs	BCL2	intronic
Chr.18:63193013	0.89	rs12457700	HRT, BLD	7 organs	19 organs	7 bound proteins	Eomes,RXRA	BCL2	intronic
Chr.18:63194460	0.87	rs4987790		BLD, MUS				BCL2	intronic
Chr.18:63200343	0.86	rs11875971	BLD	9 organs	BLD,BLD		4 altered motifs	BCL2	intronic
Chr.18:63205849	0.84	rs2062010	BLD	6 organs	BLD	EBF1,NFKB	CHD2,MAZR,SP1	BCL2	intronic

ENCODE, the Encyclopedia of DNA Elements; LD, linkage disequilibrium; Chr., Chromosome; Pos., Position

[a] LD (r^2) is based upon 1000 Genomes data and a threshold of r^2>0.8 was imposed to identify correlated SNPs
[b] Index SNPs are shown in bold.
[c] Evidence of chromatin hypersensitivity to DNase
[d] ChIP-seq experiments indicate alteration in binding of transcription factor
[e] Evidence of alteration in regulatory motif (if >3, only the number is included).

Supplementary Table 7 Functional annotation of SNPs correlated with rs13398147 (r^2 > 0.8) using data from ENCODE

Pos. (hg38)	LD (r^2)[a]	SNP[b]	Histone marks		DNAse[c]	Motifs changed[d]	genes
			Promoter	Enhancer			
Chr.2:221830626	0.83	rs7580262				Hsf	189kb 3' of AC068489.1
Chr.2:221837994	0.84	rs17312308				Pou5f1,Sox,TATA	196kb 3' of AC068489.1
Chr.2:221839640	0.84	rs62180474				GR,Pou5f1	198kb 3' of AC068489.1
Chr.2:221848813	0.84	rs72971007			BRN	9 altered motifs	207kb 3' of AC068489.1
Chr.2:221850798	0.84	rs62180482		ESDR, MUS, BRN		4 altered motifs	209kb 3' of AC068489.1
Chr.2:221851363	0.84	rs4099413		ESDR, MUS		4 altered motifs	210kb 3' of AC068489.1
Chr.2:221851517	0.84	rs4099414		ESDR, MUS	MUS	Mtf1	210kb 3' of AC068489.1
Chr.2:221853226	0.84	rs4674617				5 altered motifs	211kb 3' of AC068489.1
Chr.2:221858742	0.84	rs62180487					217kb 3' of AC068489.1
Chr.2:221861668	0.84	rs4510218	BRST			4 altered motifs	220kb 3' of AC068489.1
Chr.2:221866170	0.86	rs72971048				13 altered motifs	224kb 3' of AC068489.1
Chr.2:221873164	0.86	rs4085399				4 altered motifs	231kb 3' of AC068489.1
Chr.2:221873321	0.86	rs17312518				5 altered motifs	232kb 3' of AC068489.1
Chr.2:221873703	0.85	rs62180522				NRSF,SETDB1,Zfx	232kb 3' of AC068489.1
Chr.2:221887396	0.91	rs62180527				8 altered motifs	246kb 3' of AC068489.1
Chr.2:221891008	0.91	rs17312759					249kb 3' of AC068489.1
Chr.2:221894102	1	rs17312814		BRN		4 altered motifs	252kb 3' of AC068489.1
Chr.2:221894336	0.99	rs4674621		BRN		6 altered motifs	253kb 3' of AC068489.1
**Chr.2:221895559**	**1**	**rs13398147**		**7 organs**	**5 organs**	**5 altered motifs**	**254kb 3' of AC068489.1**
Chr.2:221896962	0.99	rs62180530	6 organs	9 organs	17 organs	BDP1,MZF1:1-4,RBP-Jkappa	255kb 3' of AC068489.1
Chr.2:221897142	0.99	rs7567306	6 organs		8 organs	Ets,GR,Rad21	255kb 3' of AC068489.1
Chr.2:221897754	0.99	rs17312891				Brachyury	256kb 3' of AC068489.1

ENCODE, the Encyclopedia of DNA Elements; LD, linkage disequilibrium; Chr., Chromosome; Pos., Position

[a] LD (r^2) is based upon 1000 Genomes data and a threshold of r^2>0.8 was imposed to identify correlated SNPs

[b] Index SNPs are shown in bold.

[c] Evidence of Chromatin hypersensitivity to DNase

[d] Evidence of alteration in regulatory motif (if >3, only the number is included).

Supplementary Table 8 Functional annotation of SNPs correlated with rs241215 (r^2 > 0.8) using data from ENCODE

Pos. (hg38)	LD (r^2)[a]	SNP[b]	Histone marks		Motifs changed[c]	genes
			Promoter	Enhancer		
Chr.1:4537029	0.96	rs472113			Mxi1,RFX5,SREBP	15kb 5' of RP1-37J18.1
Chr.1:4537941	0.97	rs608492			PLAG1,RFX5,Spz1	14kb 5' of RP1-37J18.1
Chr.1:4538975	0.94	rs148236167			9 altered motifs	13kb 5' of RP1-37J18.1
Chr.1:4539183	0.97	rs592726				13kb 5' of RP1-37J18.1
Chr.1:4540173	0.99	rs627374			Hsf	12kb 5' of RP1-37J18.1
Chr.1:4540561	0.97	rs542688			Bcl6b,NF-AT,STAT	11kb 5' of RP1-37J18.1
Chr.1:4543264	0.99	rs694935			4 altered motifs	8.5kb 5' of RP1-37J18.1
**Chr.1:4544044**	**1**	**rs241215**			**Nr2f2**	**7.7kb 5' of RP1-37J18.1**
Chr.1:4544109	1	rs241216			ERalpha-a,TAL1	7.6kb 5' of RP1-37J18.1
Chr.1:4544804	0.99	rs71580271	BRST	ESC	Foxp1,Nanog	6.9kb 5' of RP1-37J18.1
Chr.1:4548144	0.95	rs241220			4 altered motifs	3.6kb 5' of RP1-37J18.1
Chr.1:4548990	0.94	rs241221			NF-kappaB,Zfp410	2.7kb 5' of RP1-37J18.1

ENCODE, the Encyclopedia of DNA Elements; LD, linkage disequilibrium; Chr., Chromosome; Pos., Position

[a] LD (r^2) is based upon 1000 Genomes data and a threshold of r^2>0.8 was imposed to identify correlated SNPs

[b] Index SNPs are shown in bold.

[c] Evidence of alteration in regulatory motif (if >3, only the number is included).

Supplementary Table 9 Association of the 4 novel associated SNPs with curve severity of AIS patients

SNPs	Curve Severity (degrees)			P
	XX[a]	Xx	xx	
rs678741	38.1 ± 8.2	36.3 ± 9.1	37.9 ± 8.4	0.29
rs4940576	36.5 ± 8.9	36.8 ± 8.7	37.6 ± 9.1	0.37
rs13398147	37.5 ± 8.9	36.8 ± 8.3	37.4 ± 9.8	0.56
rs241215	36.7 ± 8.1	36.9 ± 7.7	39.6 ± 7.3	0.03

[a] XX, Xx, and xx indicate homozygotes with respect to the major allele, heterozygotes and homozygotes with respect to the minor allele, respectively. For rs678741, XX/Xx/xx indicates GG/GA/AA, respectively. For rs4940576, XX/Xx/xx indicates CC/CT/TT, respectively. For rs13398147, XX/Xx/xx indicates CC/CT/TT, respectively. For rs241215, XX/Xx/xx indicates TT/TA/AA, respectively.
